# Experimental Investigation of Vibration Isolator for Large Aperture Electromagnetic MEMS Micromirror

**DOI:** 10.3390/mi14081490

**Published:** 2023-07-25

**Authors:** Lei Qian, Yameng Shan, Junduo Wang, Haoxiang Li, Kewei Wang, Huijun Yu, Peng Zhou, Wenjiang Shen

**Affiliations:** 1 School of Nano-Tech and Nano-Bionics, University of Science and Technology of China, Hefei 230026, China; lei621@mail.ustc.edu.cn (L.Q.);; 2 Suzhou Institute of Nano-Tech and Nano-Bionics, Chinese Academy of Sciences, Suzhou 215123, China

**Keywords:** light detection and ranging, MEMS micromirror, vibration isolation, mechanical low-pass filter

## Abstract

The Micro-Electro-Mechanical-System (MEMS) micromirror has shown great advantages in Light Detection and Ranging (LiDAR) for autonomous vehicles. The equipment on vehicles is usually exposed to environmental vibration that may degrade or even destroy the flexure of the micromirror for its delicate structure. In this work, a mechanical low-pass filter (LPF) acting as a vibration isolator for a micromirror is proposed. The research starts with the evaluation of vibration influences on the micromirror by theoretical calculation and simulation. The results illustrate that mechanical load concentrates at the slow flexure of the micromirror as it is excited to resonate in second-order mode (named piston mode) in Z-direction vibration. A specific LPF for the micromirror is designed to attenuate the response to high-frequency vibration, especially around piston mode. The material of the LPF is a beryllium-copper alloy, chosen for its outstanding properties of elasticity, ductility, and fatigue resistance. To measure the mechanical load on the micromirror in practical, the on-chip piezoresistive sensor is utilized and a relevant test setup is built to validate the effect of the LPF. Micromirrors with or without the LPF are both tested under 10 g vibration in the Z-direction. The sensor output of the device with the LPF is 35.9 mV in piston mode, while the device without the LPF is 70.42 mV. The attenuation ratio is 0.51. This result demonstrates that the LPF structure can effectively reduce the stress caused by piston mode vibration.

## 1. Introduction

A micro-electro-mechanical-system (MEMS) is an intelligent system with electronics, optics and micromechanics integrated on a single chip in a three-dimensional structure according to functional requirements [1]. In the past decades, great progress has been made in this field, no matter in commercialization or academia, such as MEMS inertial sensors [2,3], radio frequency (RF) switches [4] and microphones [5]. Representatively, MEMS micromirrors, acting as scanners [6], have been put into diverse applications from optical cross-connect (OXC) technology [7] to projection display [8], and have achieved commercial mass production successfully in the rapid development of light detection and ranging (LiDAR) for the automotive industry in recent years [9]. Compared to a traditional motorized scanner, the MEMS micromirror is desired because it allows operating in miniaturization, on low power and at low cost, while keeping a high scanning speed and a wide field of view [10,11]. These advantages make it tremendously valuable in autonomous vehicles. Hofmann et al. proposed a 7 mm biaxial MEMS mirror for an intelligent vehicle safety system that is actuated by electrostatic combs [12]. Bo and co-workers developed a 2D micromirror with large electromagnetic driving forces to meet the demand for a sufficient scanning angular range [13].

Since MEMS devices contain a delicate movable micro-structure, they are susceptible to mechanical vibration and shock especially in an automotive environment [14]. These mechanical interferences can degrade or disturb the device’s performance. In a worse situation, the deformation response of MEMS will make microstructures fractured under a heavy environmental load [15,16]. Therefore, considerable attention should be given to the reliability of MEMS devices. Current research mainly focuses on MEMS sensors like gyroscopes [17] and accelerometers [18,19]. For environment vibration, an efficient solution is to integrate MEMS with a low-pass filter (LPF), a mass-spring-damper structure, and restrain ambient high-frequency vibration [20].

Yoon et al. presented a wafer-level design of vibration by taking gyroscopes as study object, and their experimental results showed effective suppression to over 2.1 kHz external vibration [21]. Additionally, a new metamaterial composed of phononic crystal unit-cells was utilized to fabricate a vibration isolator in Yao’s work and showed a −30 dB attenuation level in a certain range of frequency [22]. Brent et al. developed a micro-isolator fabricated from <100> silicon and investigated the transmissibility of the damped and undamped isolators by applying different fibrous meshes [23]. Regarding the MEMS scanner, Fang et al. explored the failure mechanism of an electromagnetic micromirror when it was exposed to vibration [24] and shock [25] loads, respectively, and the proposed theoretical model was in good agreement with the experimental results. These research papers offer corresponding inspiration on how to enhance the robustness. Yoo et al. evaluated the coupling vibration influence on the resonant mirror in operation that was caused by an inertial mismatch between reinforcement structure and mirror. Then, the phase-locked loop was adopted to decrease the amplitude and frequency error by way of a control algorithm [26].

From the state of the art relevant to the MEMS scanner, the three main actuation principles to be considered in vehicle LiDAR are electrostatic actuation, electromagnetic actuation, and piezoelectric actuation. Compared to the other two kinds of micromirror, the electromagnetic actuator has obvious advantages in high force and linearity to drive a large optical aperture mirror with a low voltage, as a large mirror size is required for sufficiently high resolution. However, it has disadvantages in package compactness. More importantly, the big mirror and the metal moving coil increase the mass of the movable part, which pulls the natural frequency of eigenmodes into the 20~2000 Hz band. Therefore, an environmental vibration disturbance can trigger the device to resonate in unwanted mode. This will not only impact the performance of micromirror but may even lead to device failure. 

In this paper, we take an electromagnetic micromirror as the research object, and an LPF, serving as vibration isolator, is designed to attenuate the resonance effects. In the section “Deformation analysis of micromirror”, the second mode of the micromirror, named the piston mode, is found to create a risk of damaging the micromirror’s flexure in Z-direction vibration based on FEM simulation. In the section “Design of Mechanical LPF”, the LPF is proposed to enhance the reliability of the micromirror in piston mode, which is integrated between the micromirror and the package substrate and we describe the geometric dimension and material. Simulation and theoretical analysis predict the effect of the LPF in Z-direction vibration. In the section “Experiments and Results”, we introduce the measurement method. It is always difficult to detect the slight deformation of the MEMS device, and in [21,22,23,24], the researchers apply a laser displacement meter that is costly and complex to set up. In this work, we utilize the piezoresistive sensor integrated at the flexure of the micromirror to measure the mechanical load and convert it to a voltage signal received by a data acquisition card. The operating principle of the sensor is elaborated in Section 4.1, illustrating the proportional relationship between stress and signal. Then, we verify the performance of the LPF by comparing the experimental results of the device with and without the LPF. Finally, in section “Conclusions”, we summarize our results and conclude that the LPF structure can effectively reduce the stress caused by piston mode vibration.

## 2. Deformation Analysis of Micromirror

### 2.1. Modal Analysis

**Figure 1:**
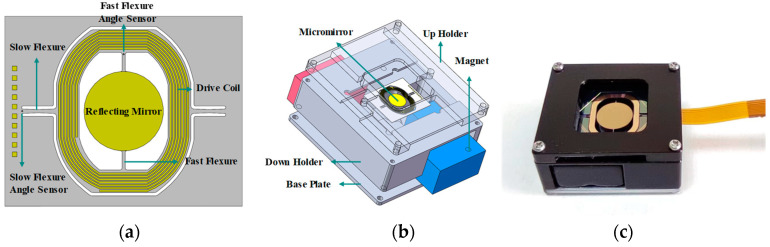
The electromagnetic MEMS micromirror and package. (**a**) Single micromirror chip. (**b**) Schematic diagram of a packaged micromirror. (**c**) Packaged MEMS micromirror.

The structure of the MEMS micromirror single chip is shown in Figure 1a. It is composed of a reflecting mirror, a metal coil gimbal, the fast flexure and the slow flexure. Moreover, the on-chip piezoresistive sensors are integrated at the end of the fast flexure and the slow flexure, respectively. The metal coil gimbal is suspended by two torsion beams, called the slow flexure, and the fast flexure extends from the gimbal connecting to the reflecting mirror vertically. The packaged MEMS micromirror is shown in Figure 1b. The package mainly includes a tube, a pedestal and two groups of permanent magnets, which provide the magnetic fields in both X and Y directions for the metal coil to generate torque when a drive current is present. The pedestal is made from steel, and the iron core at the center can enhance the magnetic induction inside the package. The tube assembles all these parts as a whole rigid body without any relative displacement. Therefore, the research focus on reliability can be simplified to the micromirror chip only.

**Figure 2:**
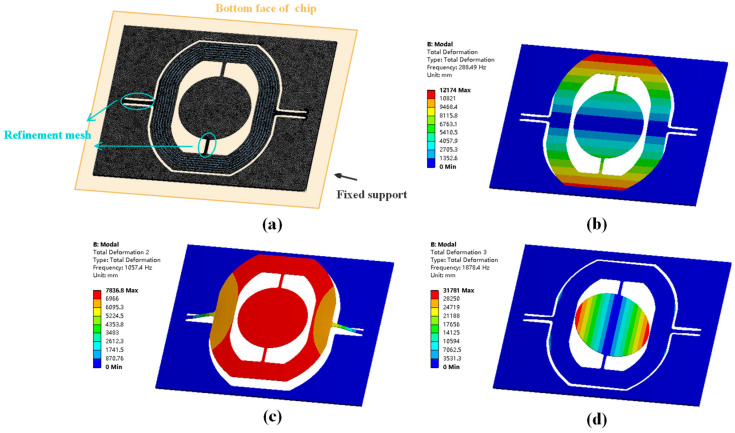
The results of modal analysis. (a) The finite model of the micromirror chip.(**b**) The first-order operation mode for the slow flexure at 288.49 Hz. (**c**) The second-order piston mode at 1057.4 Hz. (**d**) The third-order operation mode for the fast flexure at 1878.4 Hz.

According to the relevant literature, the vibration disturbance to MEMS devices in vehicles occurs in a band of 20 Hz~2000 Hz [24]. To analyze the deformation response in this frequency range, we perform simulations via the finite element method (FEM). The geometric parameters and material properties [27] used in MEMS fabrication for the micromirror are listed in Table 1. The electromagnetic MEMS micromirror is fabricated by <100> standard silicon on an insulator (SOI) wafer, and the thickness of the device layer is 55 μm. The movable part of the micromirror includes the flexures, gimbal and the mirror. The remaining part of the micromirror is called the frame part. In the modal analysis, the fixed support is applied to the bottom face of the frame part, which is the boundary condition of simulation. The finite element model is built with 127,084 elements in a mechanical module. The mesh of the model and the boundary condition are presented in Figure 2a. The flexure beams are much smaller than other parts, and they are crucial areas in the following analysis; therefore, the mesh of the slow flexure and fast flexure structure are divided by the way of refinement.

The results of modal analysis are visualized in Figure 2. There are three eigenmodes in the frequency range of 20 Hz~2000 Hz, and the first- and third-order modes are operation scanning modes for the slow flexure and the fast flexure, respectively. In the second-order mode, named the piston mode, the reflecting mirror and metal coil are moving out of the plane vertically without any rotation. The natural frequency corresponding to the piston mode is 1057 Hz. If the Z-direction vibration loads in frequency near to that value and excites the micromirror into resonance, the slow flexure would face great stress and even become fractured. 

### 2.2. Deformation Analysis

Based on modal analysis, the frequency response of deformation is conducted by means of numerical calculation and FEM simulation. As the micromirror was excited into piston mode, we can simplify the dynamic model to an equivalent mass suspended by two cantilevers, and the mass is the sum of the gimbal, metal coil and reflecting mirror. Considering the piston mode of the electromagnetically actuated micromirror as a single degree of freedom (SDoF) model [28,29]:

(1) $$m_{m}({\ddot{x}}_{m}+{\ddot{x}}_{0})+c_{m}{\dot{x}}_{m}+k_{m}x_{m}=0$$

where *m_m_* denotes the equivalent mass of the considered piston mode. *x_m_* is the deformation of the slow flexure. *c_m_* and *k_m_* represent the damping and stiffness, respectively. The acceleration waveform of vibration excitation is denoted by *x*_0′_′ = *asin*(2*πft*), where *a*, and *f* represent the amplitude and frequency, respectively. The equivalent mass value is calculated by material parameters in Table 1 and computer-aided design software. The stiffness is given by equivalent mass and the undamped natural frequency of piston mode is denoted by *f_m_*:

(2) $$f_{m}=2\pi \times \sqrt{\frac{k_{m}}{m_{m}}}$$

**Figure 3:**
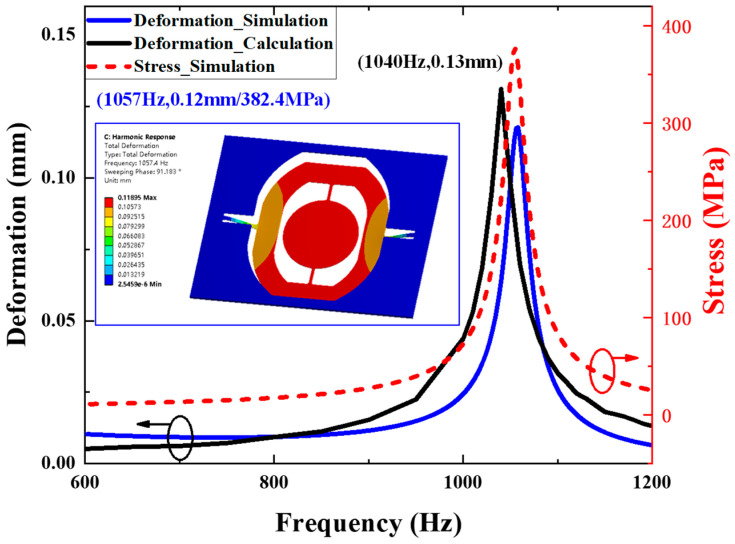
The results of frequency response by methods of numerical calculation and FEM simulation. Black circle notes Deformation_Simulation and Deformation_Calculation curves to left Y-axis and red circle notes Stress_Simulation curve to right Y-axis.

Numerical calculation was conducted in the MATLAB software. To obtain the deformation response of slow flexure to different vibration frequencies, series values of frequency *f* are substituted into Equation (1) and solve these equations. The value of *f* varies in intervals of [600 Hz, 1200 Hz], which is around the piston mode according to the results of modal analysis. The results are exhibited in Figure 3. When the excitation level is fixed at 10 g and the frequency varies around the piston mode, the deformation of the slow flexure increases first and then decreases with rising frequency. The deformation reaches a maximum of 0.13 mm at 1040 Hz, regarded as the resonance state, as described in Figure 2b. Moreover, FEM simulation of harmonic response was performed linked from modal analysis as well. The inertial excitation was set to all fixed supports, and the acceleration was 10 g as well. According to Figure 3, the results show good agreement with numerical calculation. The response curve exhibits a typical resonance feature; the deformation peak occurs at 1057 Hz, and the slow flexure stretches to 0.12 mm with a maximum stress of 382.4 MPa. The deformation of the micromirror is shown in the embedded figure.

## 3. Design of Mechanical LPF

### 3.1. Principle

**Figure 4:**
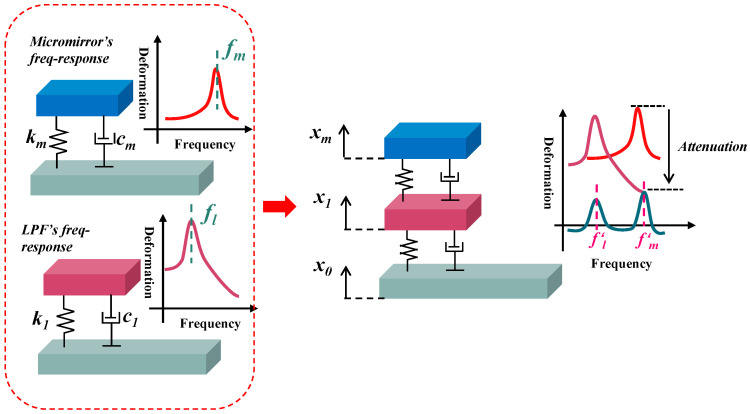
Operation of mechanical low-pass filter integrated with MEMS micromirror.

Figure 4 conceptually presents the principle of the mechanical low-pass filter that serves as a vibration isolator. In essence, the LPF is a set of mass-spring-damper assembly that is inserted between the micromirror and package substrate. 

It is explicit that the micromirror has a large deformation under the vibration with frequency around the piston mode (*f_m_*). By adjusting the stiffness of the LPF, the natural frequency (*f_l_*) can be set to be low enough, which should be smaller than *f_m_*. According to the physics rule of resonance, the further away from the natural frequency, the smaller the amplitude caused by the vibration. Therefore, the displacement of the LPF reaches a relative tiny level when the vibration frequency is much higher than *f_l_*, and it then attenuates the response amplitude of micromirror in piston mode.

The attenuation extent depends on the value of *f_l_*, the LPF’s quality factor (Q) and the attenuation slope. In practical application, the filter’s resonance frequency is designed to be smaller than the band desired to be suppressed or the frequency that sensors are susceptible to, and the larger the difference, the more significant the attenuation level will be. A high-Q filter is able to achieve a steep slope to obtain the ideal attenuation level quickly; however, it may cause the amplitude of the filter itself to be exceeded, which is harmful to the target device as well [23]. Therefore, it is a tradeoff in filter design, as low Q cannot meet suppression requirements. In addition, multi-order isolators can be used to improve the filter performance, which means increasing the number of LPFs. 

Accordingly, the dynamic equations of a two-degree freedom model are analyzed, where *x*_1_ represents the deformation of the LPF spring and the damping and stiffness are denoted by *c*_1_, *k*_1_. The remaining parameters are explained as before.

Motion equations are given by [30]:

(3) $$m_{m}\left({\ddot{x}}_{m}+{\ddot{x}}_{1}+{\ddot{x}}_{0}\right)=-c_{m}{\dot{x}}_{m}-k_{m}x_{m}$$

(4) $$m_{1}\left({\ddot{x}}_{1}+{\ddot{x}}_{0}\right)=c_{m}{\dot{x}}_{m}+k_{d}x_{d}-c_{1}{\dot{x}}_{1}-x_{1}$$

(5) $${\ddot{x}}_{0}=asin(2\pi ft)$$

These three equations involve three unknown variables, *x*_0_, *x*_1_, *x_m_*, forming a set of differential equations. As shown in Figure 4, *x*_0_ represents the absolute displacement of excitation, the shake table for example, and its acceleration varies as a sine function, described in Equation (5). Equations (3) and (4) correspond to the micromirror and the LPF, respectively, and *x_m_* is the deformation of the micromirror’s slow flexure. Compared to the single-degree-of-freedom model, there are some cross terms in equations, which will change the response amplitude to vibration. By solving the equations group, the variation of *x*_1_ and *x_m_* are made clear, as explained in the following text.

### 3.2. Structure Design

**Figure 5:**
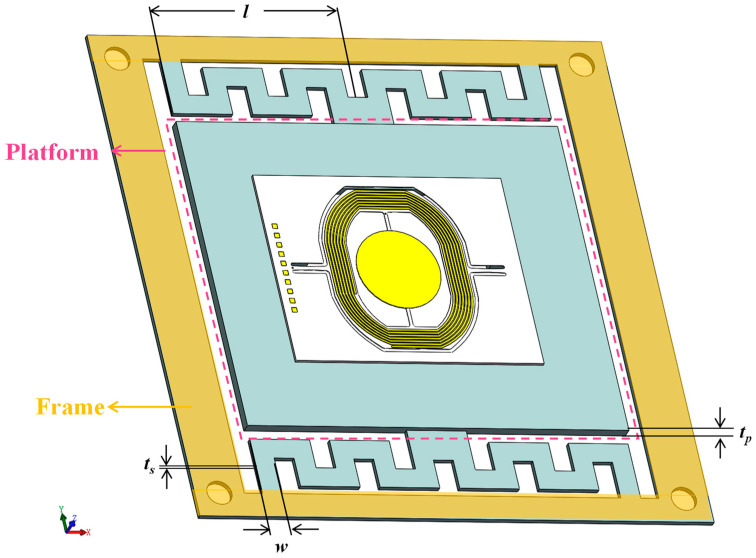
Schematic diagram of the mechanical low-pass filter integrated with a micromirror.

The proposed LPF, according to the micromirror’s resonance frequency, is shown in Figure 5. The structure applies a folded beam as a suspension spring, and the stiffness is determined by the out-of-plane thickness *t_s_*, width *w* and length of the beam *l*. The thickness of the platform *t_p_* is designed to be more than the folder beam to prevent the shape change of the platform from generating extra stress on the micromirror. The frame is the area bonded to the package substrate. These main geometric parameters are listed in Table 2. The overall dimension is 33 × 33 mm. In terms of material, the literature on vibration isolators on MEMS devices focuses on single-crystal silicon for its advantages, such as great compatibility with standard MEMS fabrication process and high precision of dimensions to realize a delicate design [20,21,22,23,24]. Nevertheless, silicon is still imperfect when it comes to large stretching, as a brittle material [31]. It is important to note that the resonance frequency of the LPF is intentionally set to be low enough to ensure adequate filter performance, which means small stiffness and large dynamic deformation, relatively. Therefore, a silicon-based LPF is at greater risk of being fractured. By contrast, metal is more ductile and possesses a high breaking strength, which meets the reliability requirement. In this research, we choose a beryllium–copper alloy to make the LPF for its outstanding properties of elasticity, fatigue resistance and fracture strength [32]. The beryllium–copper alloy’s yield strength is up to 1110 MPa and it is non-magnetic [33], making it suitable to apply in this case.

### 3.3. Deformation Analysis of Micromirror with LPF

**Figure 6:**
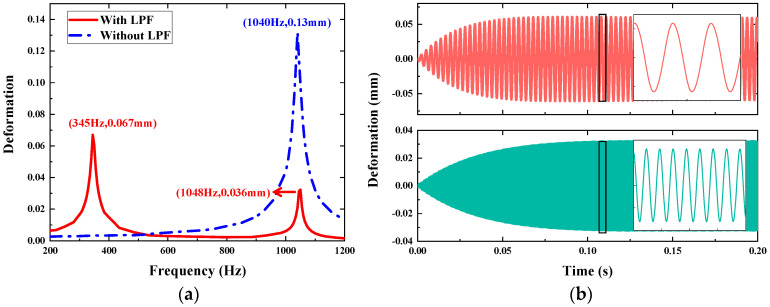
Results of the micromirror’s slow flexure deformation under 10 g vertical vibration by numerical calculation. (**a**) Frequency response. (**b**) Transient response at two resonance states. Red graph (**top**) represents response at 345 Hz and green graph (**bottom**) represents response at 1048 Hz.

In this section, the micromirror integrated with the LPF is analyzed as an assembly, with a particular focus on the deformation of the slow flexure and the spring of the LPF. Similarly, we investigate the response to vibration by approaches of FEM simulation and numerical calculation. In the numerical calculation, excitation frequency ω is set to vary around the micromirror and LPF resonance in Equation (5). To obtain the frequency response curve of the integral structure, the differential equations are solved by combining Equations (3) and (4). The numerical calculation results of the device with and without the LPF are compared in Figure 6a, and the deformation in piston mode is obviously attenuated from 0.13 mm to 0.036 mm. The other deformation peak occurs at LPF resonance as 0.067 mm. Figure 6b describes the transient responses of the micromirror’s slow flexure at 345 Hz and 1048 Hz, respectively.

In the FEM simulation, the following material parameters of the beryllium–copper alloy are used: density is 8.3 × 10^3^ kg/m^3^, Young’s modulus is 110 GPa and Poisson’s ratio is 0.34. The bottom surface of the micromirror is set to be bonded to the top surface of the LFP without any relative displacement. The fixed supports are applied to the bottom surface of the LPF’s frame, and in harmonic response analysis, the applied load is 10 g acceleration in the Z-direction vertically. 

**Figure 7:**
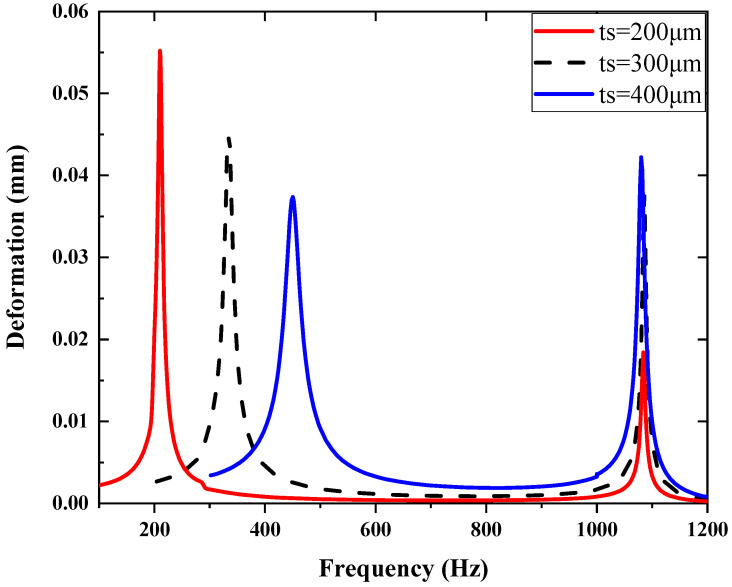
The influence of different thicknesses of LPF on micromirror.

In parametric analysis, the overall size is fixed to fit the device package, so the LPF is optimized by changing the thickness of the LPF spring (*t_s_*) to adjust the stiffness. As shown in Figure 7, simulation is conducted to observe the influence of different thicknesses on the micromirror. The value of the Y-axis represents the deformation of the micromirror’s slow flexure. With the thickness increasing, the natural frequency of the LPF increases as the stiffness becomes harder. The deformation of the micromirror at the low-frequency band is smaller with a harder LPF applied, but the attenuation in piston mode of the micromirror becomes weak, which is in agreement with the discussion in Section 3.1. If the stiffness is designed to be too low, it would cause a large mechanical load on the micromirror and the LPF, although it achieves good attenuation in piston mode. Therefore, it is a tradeoff in design to realize a good isolation effect while keeping low deformation in the low-frequency band. In this work, the thickness of 300 μm is preferred for its appropriate performance across the full frequency band.

**Figure 8:**
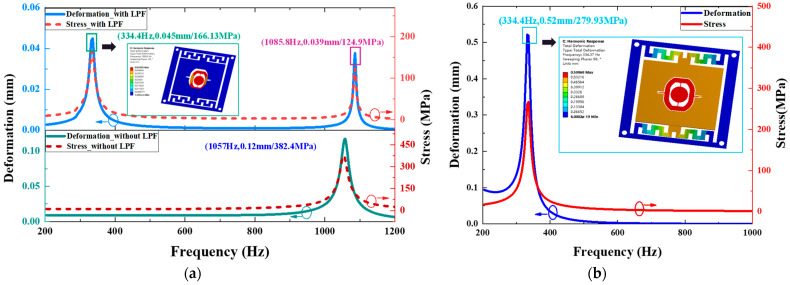
Simulation results of micromirror with LPF under sweeping frequency excitation. (**a**) The deformation and maximum stress of the micromirror’s slow flexure. (**b**) The deformation and maximum stress of the LPF spring. Blue circle notes blue Deformation curve to left Y-axis and red circle notes red Stress curve to right Y-axis.

In Figure 8, the plots describe the deformation and stress of the LPF spring and the micromirror’s slow flexure, compared to the device without the LPF. As shown in Figure 8a, the deformation of the slow flexure reaches two peak values of 0.045 mm and 0.039 mm at 334.4 Hz and 1085.8 Hz, respectively. This is close to the results of numerical calculation. The corresponding stress values are 166.3 MPa and 124.9 MPa. The total deformation distribution cloud picture at 334.4 Hz is embedded in Figure 7a. It exhibits the attenuation effects according to the simulation results. The total deformation distribution of harmonic response analyses at 334.4 Hz and 1085.8 Hz are presented in Figure 7b. The deformation LPF reaches the peak value of 0.52 mm at 334.4 Hz with maximum stress of 279.93 MPa, as shown in Figure 8b, and the total deformation distribution is shown in the embedded picture. The maximum stress value of the LPF is far smaller than the yield strength of the beryllium–copper alloy.

## 4. Experiments and Results

In this section, experiments are conducted to verify the effect of the LPF under vibration excitation. The piezoresistive sensor is utilized to measure the load of the micromirror’s slow flexure. The sample devices with and without the LPF are tested and compared. 

### 4.1. Piezoresistive Sense Principle

**Figure 9:**
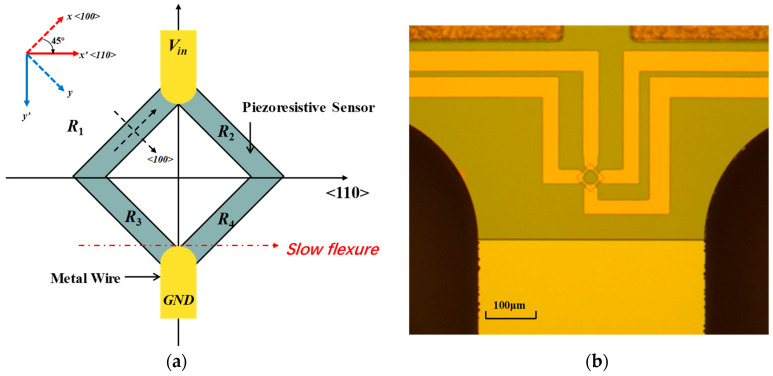
(**a**) The diagram of piezoresistive sensor configuration and silicon crystal coordination. (**b**) Picture of piezoresistive sensor under a microscope.

The piezoresistive sensor is integrated at the end of the rotation flexures by ion implantation, as shown in Figure 9b, to detect the deflection angle in working states. The sensor is configured in the form of a diamond, with four resistors connected, and made to be a Wheatstone bridge to measure the stress caused by torsion. In this research, the goal of the experiments is to measure and compare the stretch of the micromirror’s slow flexure with or without the LPF. 

The piezoresistive effect in single-crystal silicon can be described by the well-known equation

(6) $$\left[\begin{array}{c} \Delta \rho_{1}\\ \Delta \rho_{2}\\ \Delta \rho_{3}\\ \Delta \rho_{4}\\ \Delta \rho_{5}\\ \Delta \rho_{6} \end{array}\right]=\rho_{0}\left[\begin{array}{cccccc} \pi_{11} & \pi_{12} & \pi_{12} & 0 & 0 & 0\\ \pi_{12} & \pi_{11} & \pi_{12} & 0 & 0 & 0\\ \pi_{12} & \pi_{12} & \pi_{11} & 0 & 0 & 0\\ 0 & 0 & 0 & \pi_{44} & 0 & 0\\ 0 & 0 & 0 & 0 & \pi_{44} & 0\\ 0 & 0 & 0 & 0 & 0 & \pi_{44} \end{array}\right]\left[\begin{array}{c} \sigma_{1}\\ \sigma_{2}\\ \sigma_{3}\\ \sigma_{4}\\ \sigma_{5}\\ \sigma_{6} \end{array}\right]$$

where *π*_11_, *π*_12_, and *π*_44_ are longitudinal, transverse and sheer piezoresistive coefficients, respectively. *σ_i_* represents the stress tensor with i = 1, 2, 3 denoting axial stress and I = 4, 5, 6 denoting sheer stress. For a thin piezoresistor, the piezoresistive effect mainly produced by axial stress in the X- and Y-directions, sheer stress in the X–Y plane and sheer stress in the remaining plane are too small and therefore negligible [34]. Thus, Equation (6) approximates to

(7) $$\frac{∆\rho }{\rho }=\frac{∆R}{R}={\pi^{\prime }}_{11}\sigma_{1}+{\pi^{\prime }}_{12}\sigma_{2}+{\pi^{\prime }}_{16}\sigma_{6}$$

The piezoresistive coefficient needs to be transformed from the silicon crystal graphic coordinate system into the coordinate system, the same as with the stress tensor. The transformation to [*π*] is performed by

(8) $$\pi^{\prime }=R\pi R^{-1}$$

where *R* is [35]:

(9) $$\left[\begin{array}{cccccc} l_{1}^{2} & m_{1}^{2} & n_{1}^{2} & 2m_{1}n_{1} & 2l_{1}n_{1} & 2l_{1}m_{1}\\ l_{2}^{2} & m_{2}^{2} & n_{2}^{2} & 2m_{2}n_{2} & 2l_{2}n_{2} & 2l_{2}m_{2}\\ l_{3}^{2} & m_{3}^{2} & n_{3}^{2} & 2m_{3}n_{3} & 2l_{3}n_{3} & 2l_{3}m_{3}\\ l_{2}l_{3} & m_{2}m_{3} & n_{2}n_{3} & m_{2}n_{3}+m_{2}n_{3} & l_{2}n_{3}+l_{3}n_{2} & m_{2}l_{3}+m_{3}l_{2}\\ l_{1}l_{3} & m_{1}m_{3} & n_{1}n_{3} & m_{1}n_{3}+m_{3}n_{1} & l_{1}n_{3}+l_{3}n_{1} & m_{1}l_{3}+m_{3}l_{1}\\ l_{1}l_{2} & m_{1}m_{2} & n_{1}n_{2} & m_{1}n_{2}+m_{2}n_{1} & l_{1}n_{2}+l_{2}n_{1} & m_{1}l_{2}+m_{2}l_{1} \end{array}\right]$$

The element in matrix *R* is the direction cosines of the coordinate system. In this research, the silicon wafer used in micromirror fabrication is a standard (100) wafer, and the slow flexure is aligned in the <110> silicon direction, as shown in Figure 9a. Therefore, the direction cosines are

(10) $$\left[\begin{array}{ccc} l_{1} & m_{1} & n_{1}\\ l_{2} & m_{2} & n_{2}\\ l_{3} & m_{3} & n_{3} \end{array}\right]=\left[\begin{array}{ccc} \frac{1}{\sqrt{2}} & \frac{1}{\sqrt{2}} & 0\\ -\frac{1}{\sqrt{2}} & \frac{1}{\sqrt{2}} & 0\\ 0 & 0 & 1 \end{array}\right]$$

Substituting Equations (8)–(10) into Equation (7), we can obtain the resistance change of one single piezoresistor:

(11) $$\frac{∆R}{R}=\frac{1}{2}\left(\sigma_{1}+\sigma_{1}\right)\left(\pi_{11}+\pi_{12}\right)+\sigma_{6}\left(\pi_{11}-\pi_{12}\right)$$

Accordingly, the stress will change the resistance of the sensor and convert to a voltage signal with DC bias. In working mode, the flexure is mainly subject to shear stress *σ_6_* for torsion motion, which can be used to detect the scanning angle. While in piston mode, the flexure is mainly subject to axial stress *σ_1_* or *σ_2_* for stretch strain. All these mechanical loads are reflected in Equation (11). In the following experiments, only the piston mode would be triggered in a particular frequency excitation. 

### 4.2. Experimental Setup

**Figure 10:**
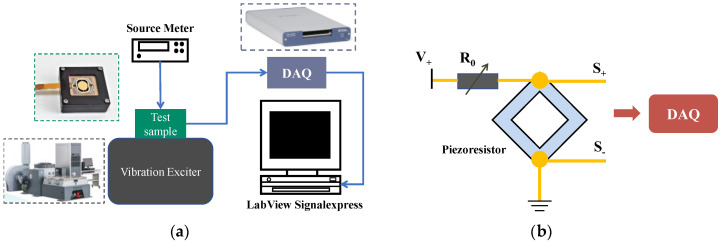
(**a**) Experimental setup using vibration exciter. (**b**) Single end measurement circuit diagram; the R_0_ is an out-of-chip resistor acting as a zero setting.

The experiment setup is shown in Figure 10a, and the single-end measurement circuit is shown in Figure 10b. The device being tested is bonded on the shaker table controlled by a custom terminal, which can supply excitation signals with constant or sweeping frequencies. The magnitude of vibration acceleration is 10 g to test the device. To capture the stress load on the micromirror’s slow flexure, a voltage divider circuit is established. The input voltage is 6 V using a source meter and the divide resistor is of the same value as the piezoresistive sensor’s stress-free resistance to build a 3 V DC bias. The output signal of the piezoresistive sensor is imported to the data acquisition card (NI 6361) with the sampling frequency of 100 kHz, and the waveform data are plotted and stored in a LabVIEW program. Data processing is finally performed by MATLAB software. All the connections to the device chip under test are switched by a flexible flat cable (FPC), which is linked to the pads on the chip by wire bonding. As discussed before, the piezoresistive sensor integrated on the chip can detect the mechanical load on the flexure, so the output signal of the device, under the same vibration level, with or without the LPF can be compared to verify the attenuation effect of the LPF. The attenuation ratio is

(12) $$A=\frac{S_{with}}{S_{without}}$$

where *S* represents the peak-to-peak amplitude of the signal waveform.

### 4.3. Results

**Figure 11:**
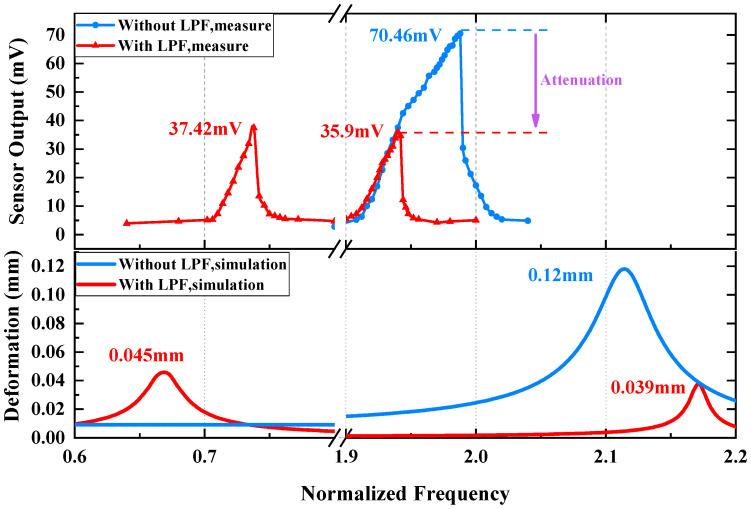
The test results of frequency response to micromirror with and without LFP, with comparison to simulation results.

There are two parts in the experiments to evaluate the effect of the LPF for the micromirror, one of which is the frequency response test, and the other is the harmonic response test. In the frequency response test, the steady-state vibration response of the micromirror’s mechanical load to different single-tone vibrations is measured and a set of envelope amplitudes of response waveforms is recorded and plotted. The frequency response in Figure 11 illustrates the behavior of the micromirror with or without the LPF, with a comparison to simulation results. For the device without the LPF, when the frequency increases to more than 960 Hz, the output of the piezoresistive sensor rises gradually and a peak value is observed when the sweeping frequency reaches 994 Hz, which is attributed to the resonance of the piston mode analyzed in Section 2.1. The measured resonance frequency is slightly lower than the value predicted by FEM and numerical calculation. For the device with the LPF, the frequency response exhibits two resonance peaks with the frequency at 369 Hz and 970 Hz, corresponding to the resonance of the LPF and micromirror’s piston mode retained, respectively. It is important to note a significantly reduced peak value at high resonant frequency, which proves the attenuation effect of the LPF on the micromirror’s piston mode. However, there is still a relatively large response at low resonant frequency caused by the LPF, despite it being smaller than the maximum response of the micromirror without the LPF. 

**Figure 12:**
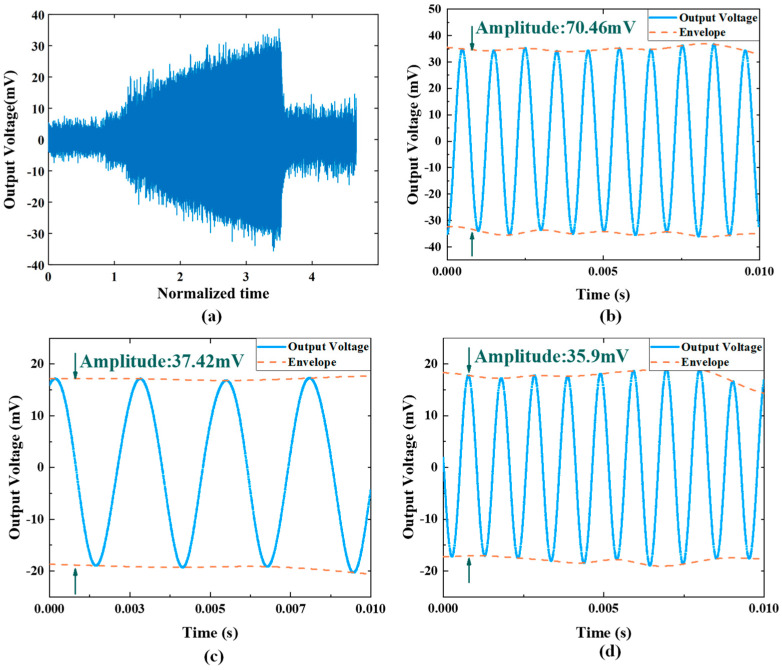
The test results of harmonic response. (**a**) Real-time response of micromirror without LPF under rising frequency vibration. (**b**) Harmonic response of micromirror without LPF at 994 Hz. (**c**) Harmonic response of micromirror with LPF at 369 Hz. (**d**) Harmonic response of micromirror with LPF at 970 Hz.

In the harmonic response test, the shaker table provides continuous sweeping frequency vibration around the devices’ resonance, and the time domain data are captured to observe transient response. Figure 12 describes the transient response of oscillating to resonance. As shown in Figure 12a, the vibration frequency of the shaker table is set to sweep around the piston mode within a period of time, and the mechanical load of the micromirror without the LPF significantly increases when the vibration frequency is greater than a certain frequency. Figure 12b exhibits the harmonic response of the micromirror without the LPF at 994 Hz, and the sensor output is 70.46 mV. Figure 12c,d depict the harmonic response of the micromirror with the LPF to high-frequency resonance and low frequency resonance. In the low-frequency band, the micromirror reaches the maximum amplitude of 37.42 mV. In the high-frequency band, the sensor output is 35.9 mV. Substituting the results into Equation (12), the attenuation ratio at the micromirror’s piston mode is 0.51. 

In the experimental part, frequency response tests and harmonic response tests are conducted to validate the performance of the LPF. For the micromirror without the LPF, the maximum deformation only occurs at 994 Hz, caused by the piston mode. With the LPF applied, the mechanical load on the micromirror’s slow flexure emerges as an extra resonance peak at 369 Hz caused by the LPF; moreover, the piston mode remained. This phenomenon is predicted by simulation and numerical calculation. Compared with the device without the LPF, the device with the LPF has amplitudes smaller than the original amplitude in both resonant frequency ranges, indicating that the LPF structure can effectively reduce the stress caused by piston mode vibration.

## 5. Conclusions

This paper presents analyses and experimental results of a mechanical low-pass filter that serves as a vibration isolator applied to a MEMS electromagnetic micromirror for automotive LiDAR. In practical application, the micromirror on vehicle is seriously influenced by vibration disturbances, which not only interfere with the performance of the integrated on-chip angle sensor, but also raise the potential risk of device damage. Against this background, simulation on FEM and numerical calculations are conducted to study the deformation response to vibration. Apart from the operating mode, the piston mode is found to lead to large deformation and stress on the slow flexure in Z-direction vibration. To improve the reliability in piston mode, a mechanical low-pass filter for the micromirror is proposed. FEM simulation and a mathematic model are established to predict the response to external vibration. A beryllium–copper alloy is chosen for its high ductility. To verify the effect of the LPF, an on-chip piezoresistive sensor is utilized to test the mechanical load of the slow flexure. The magnitude of vibration excitation is uniformly set as 10 g. For the micromirror without the LPF, the amplitude of sensor output reaches the maximum value of 70.46 mV at 994 Hz. For the micromirror with the LPF, there are two resonance responses corresponding to the LPF structure and piston mode of the micromirror, and the peak values of the sensor’s output amplitude are 37.42 mV at 369 Hz and 35.9 mV at 970 Hz, respectively. The attenuation ratio is 0.51 in piston mode. The experimental results are fairly close to the simulation results. The proposed design provides considerable attenuation for the micromirror at low cost, which relieves the stress on the slow flexure, as expected. 

However, based on experiment results, there is still a relatively large mechanical load when the vibration occurs around the LPF’s resonant frequency. Therefore, it may degrade the reliability of the micromirror at low-frequency disturbance. Furthermore, future work will focus on attenuating the deformation at low frequency caused by the LPF to realize isolation across the full frequency band, and we will implement this by optimizing the structural design and material selection. In addition, shock protection is an important research subject in studies of MEMS reliability as well, and we intend to carry out the investigation into the shock protection of micromirrors for vehicle LiDAR.

## Figures and Tables

**Table 1:** Geometric parameters and material properties.

Parameters	Value
Width of slow flexure	210 μm
Length of slow flexure	1980 μm
Thickness of slow flexure	55 μm
Density of silicon	2.33 × 10^3^ Kg/m^3^
Density of gold	19.3 × 10^3^ Kg/m^3^
Young’s modulus of silicon	130 GPa
Poisson’s ratio of silicon	0.28
Shear modulus of silicon	79.6 GPa

**Table 2:** Geometric parameters of LPF.

Parameters	Value
Width of beam	2.5 mm
Length of beam	13.75 mm
Thickness of beam	300 μm
Thickness of platform	600 μm
Mass of equivalent mass	1.6 g

## Data Availability

The data sharing in this study is not applicable.
